# Effects of biochar-based materials on nickel adsorption and bioavailability in soil

**DOI:** 10.1038/s41598-023-32502-x

**Published:** 2023-04-11

**Authors:** Weichun Gao, Wei He, Jun Zhang, Yifei Chen, Zhaoxin Zhang, Yuxiao Yang, Zhenjia He

**Affiliations:** 1 Shaanxi Provincial Land Engineering Construction Group Co., Ltd., Xi’an, 710075 China; 2 School of Water Resources and Hydropower, Xi’an University of Technology, Xi’an, 710048 China; 3 Institute of Land Engineering and Technology, Shaanxi Provincial Land Engineering Construction Group Co., Ltd., Xi’an, 710075 China; 4 College of Chemistry and Chemical Engineering, Shaanxi Key Research Laboratory of Chemical Additives, Shaanxi University of Science and Technology, Xi’an, 710021 China

**Keywords:** Environmental sciences, Chemistry

## Abstract

The potential for toxic elements to contaminate soil has been extensively studied. Therefore, the development of cost-effective methods and materials to prevent toxic element residues in the soil from entering the food chain is of great significance. Industrial and agricultural wastes such as wood vinegar (WV), sodium humate (NaHA) and biochar (BC) were used as raw materials in this study. HA was obtained by acidizing NaHA with WV and then loaded onto BC, which successfully prepared a highly efficient modification agent for nickel-contaminated soil, namely biochar-humic acid material (BC-HA). The characteristics and parameters of BC-HA were obtained by FTIR, SEM, EDS, BET and XPS. The chemisorption of Ni(II) ions by BC-HA conforms to the quasi-second-order kinetic model. Ni(II) ions are distributed on the heterogeneous surface of BC-HA by multimolecular layer adsorption, which accords with the Freundlich isotherm model. WV promotes better binding of HA and BC by introducing more active sites, thus increasing the adsorption capacity of Ni(II) ions on BC-HA. Ni(II) ions in soil are anchored to BC-HA by physical and chemical adsorption, electrostatic interaction, ion exchange and synergy.

## Introduction

With the rapid development of industrialization, urbanization and intensification of agricultural production in China, the situation of Potentially toxic elements (PTEs) pollution in farmland soil is becoming a serious health and environmental challenge^1^. Human exposure to nickel in soil depends on nickel concentration and bioavailability^2^. Agricultural soils are prone to accumulation of PTEs, which pose a major threat to food security due to their flow and transfer in the food chain^3^. Nickel is a transition element widely distributed in air, water and soil, and it exists most widely in biological systems and environments in the divalent oxidation state^4^. Fertilizers, pesticides, mining smelting and electroplating industries are the main sources of nickel^5^. At very low levels, nickel is an essential micronutrient; When the content is high, it has adverse effects on photosynthesis, plant growth, quality and yield of plants^6^. Nickel and nickel compounds accumulated in the human body pose potential health risks to human health, such as pulmonary fibrosis, renal dysfunction, cardiovascular disease and respiratory cancer^7^. The toxic element Nickel in soil not only causes serious ecological and environmental problems, but also poses a major threat to the health of organisms including human beings. Therefore, it is very important to develop efficient methods to solve the problem of nickel contaminated soil^8^.

PTEs pollution led to the aggravation of land degradation and the gradual reduction of production land. Therefore, researchers at home and abroad continue to increase their enthusiasm for soil remediation^9^. Fixed PTEs means to stabilize or reduce their mobility in the soil, with the aim of reducing the bioavailability of plants, animals and humans and reducing the leaching of PTEs to groundwater^10^. Over the past few decades, a variety of remediation methods for PTEs contaminated soils have been investigated, including soil washing^11^, electric remediation^12^, solidification/stabilization^13^, phytoremediation and biological/microbial treatment^14^, vitrification landfill and bioreactor to reduce heavy metal bioavailability^15^. Among these methods, cleaning the contaminated soil was difficult and time-consuming, so there was a need to develop techniques to fix heavy metals in the soil before returning it to agricultural use. Soil amendments not only act as immobilization agents for PTEs, but also improve plant growth and soil health by providing nutrients, improving soil structure, and enhancing holding capacity^16^. Immobilization of PETs with low-cost, highly efficient soil amendments is a promising approach to cost-effective and environmentally sound remediation^17^.

Fertilizers, fly ash and lime materials, biochar, clay minerals, compost, phosphate compounds and other soil conditioners have been widely used as soil PTEs fixative materials, among which biochar^18^ and humic acid^19^ are used as soil conditioners have received much attention. Biochar is increasingly being tested for its value in immobilizing PTEs^20^. Biochar has high pH value, aromatic carbon content and cation exchange capacity, and PTEs in soil were fixed by biochar by means of complex reaction, physical adsorption, redox, electrostatic interaction and ion exchange, so the biological utilization of PTEs in soil was greatly reduced^21^. In order to maximize the ability of biochar to adsorb PTEs, the biochar was properly modified, and the treated composite modifier had stronger stabilization/fixation ability than the single modifier. For example, biochar from sludge pyrolysis was used as soil amendment to fix lead and cadmium in soil around lead–zinc mines^22^. The combination of biochar and compost achieved good results in remediation of soil PTEs (especially Cd and Zn) in wetland soils compared with the control group^23^. The bioavailability of cadmium in paddy fields was significantly reduced after adding a mixed amendment of straw biochar and apatite ore^24^. Some studies have found that the mixture of biochar and apatite generates a lot of negative charges on the soil surface, which was conducive to the adsorption of Pb, Cd and Zn ions, resulting in a greatly reduced exchangeable content of PTEs^25^. The use of amorphous MnO_2_ modified biochar to remove heavy metal pollutants in water was also a good method for the treatment of heavy metal contaminated wastewater^26^. The combined application of biochar and acidifying manure significantly reduced the uptake and bioavailability of Cr by plants in the studied soils^27^. The combined application of biochar and phosphate showed better fixation capacity in polymetallic-contaminated soils compared with the application of biochar alone^28^. Compared with biochar (BC) and mussel shells (MS) alone, the two complexes exhibited higher Ni immobilization efficiency in contaminated soil, significantly reducing the availability and toxicity of Ni ions to crops and plants^17^. Humic and fulvic acids present in dissolved organic matter in soils play a major role in controlling the dynamics and bioavailability of nutrients and PTEs in soils^29^.

Humic acid is a natural metal chelator, which affects and controls the transport of metals through various functions and affects the absorption of metals to plants^30^. The interaction between humic acid and metal ions induces poorly soluble humic acid-metal complexes, thereby affecting the colloidal aggregation of humic acid and the migration of toxic metals^31^. They confirmed that the carboxyl and hydroxyl groups in humic acid play important roles in the interaction with toxic metal ions^32^. The active oxygen-containing functional groups of biochar powder and humic acid combine with metal cations to form metal complexes, which lead to the passivation of PTEs in the soil and will not cause secondary pollution to the soil, so they are the preferred PTEs curing agents. In addition, wood vinegar (WV) is mainly an organic acid, and its closely related organic substances can be used to complex metals and reduce the mobility of metals^33^. It has been reported that the organic acids (hydroxyl and carboxyl) contained in wood vinegar have many metal chelation adsorption sites, and have been used as good additives for the removal of pollutants such as copper, cadmium and nickel^34^. Wood vinegar has been used to compost solid waste to adsorb and immobilize metal contaminants. Wood vinegar can change the structure of organic compounds and form insoluble metal complexes during composting, thus reducing the bioavailability of metals^35^. Industrial and agricultural wastes such as wood vinegar (WV), sodium humate (NaHA) and biochar (BC) were used as raw materials. HA was obtained by acidizing NaHA with WV and then loaded onto BC, which successfully prepared a highly efficient modification agent for nickel-contaminated soil, namely biochar-humic acid material (BC-HA). The application of biochar-humic acid materials to the project of remediation of nickel-contaminated soil has formed the ecological and economic concept of “waste recycling-green composite-soil improvement”, which provides a novel design idea for the remediation and treatment of PTEs contaminated soil. This method can not only replace chemical reagents to reduce the re-harm to the soil in the process of restoration and weaken the deposition effect of PTEs in the soil, but also play a role in improving the soil structure and enhancing the fertility, which has a broad market prospect and huge environmental benefits.

This work aims to study the effect of biochar-humic acid material (BC-HA) on the available content of nickel in soil and the distribution of four chemical forms of nickel, and further evaluate the migration law and bioavailability of nickel in soil. In this experiment, the effects of adsorption kinetics, adsorption thermodynamics and pH value on the adsorption of Ni(II) by BC-HA were also investigated. Fourier transform infrared spectroscopy (FT-IR), Scanning electron spectroscopy (SEM–EDS), Surface area and porosity analyzer (BET) and X-ray photoelectron spectroscopy (XPS) were used to study the adsorption mechanism of Ni(II) in soil by biochar-based materials.

## Experimental

### Preparation of soil samples

The 0–20 cm surface soil was taken from the agricultural land in Xi’an, Shaanxi province, china. After the soil samples were air-dried, rocks, debris and animal and plant remains were removed through a 0.15 mm sieve and used as original soil samples. The pH of the original soil was 7.96, the value of organic matter was 14.82 g/kg, the value of CEC was 17.34 cmol/kg, the exchange acid was 54.30 mmol/kg and the background concentration of nickel was 12.88 mg/kg.

In this experiment, the laboratory artificially simulated nickel-contaminated soil. According to the soil pollution risk screening value of agricultural land (GB15618-2018, China), when pH > 7.5, the risk screening value of nickel is 190 mg/kg. Therefore, the pollution value is set to 200 mg/kg nickel solution. 1 kg of original soil was polluted with Ni(NO_3_)_2·_6·H_2_O (containing 201.86 mg/kg Ni^2+^). Each 1 kg air-dried experimental soil sample was evenly mixed with the remediation agent, and the contaminated soil treated with no remediation materials was used as the control group. These treatments are placed in 1.5 L plastic pots (15 cm in diameter and 13 cm in height) and irrigated to reach 60% of their water-holding capacity. These pots were exposed to greenhouse conditions at 25/18 °C (day/night), and the pots were weighed every five days and supplemented with evaporated water to maintain moisture content. Contaminated soil samples are aged for 60 days.

### Materials

Biochar was purchased from Xianyang Chemical Industry Co., Ltd., Shaanxi Province (Xianyang, China). The straw biochar obtained by the oxygen-limited pyrolysis carbonization technology was ground and passed through a 0.15 mm sieve for use. Sodium humate, a dark brown powder produced from lignite as raw material, was purchased from Shandong Zibo Hongtong Chemical Co., Ltd. (Zibo, China). The water solubility of sodium humate is not less than 85%, the pH value is 10.85, the density is 0.73 kg·L^−1^, and it is ground and passed through a 0.125 mm sieve for use. Wood vinegar liquid was purchased from Shanxi Province Jinan Xinyi Chemical Co., Ltd. (Jinan, China), a waste liquid produced in the charcoal manufacturing process of Chinese oak. The waste liquid was left standing for 5 days in the laboratory, filtered and distilled, and the fractions at a temperature of 120–150 °C were collected, with a pH value of 3.74 and a specific gravity of 0.996 g·mL^−1^.

Nickel nitrate hexahydrate (AR grade), acetic acid (AR grade), and ammonium acetate (AR grade), purchased from Shanghai McLean Biochemical Co., Ltd. (Shanghai, China). Nitric acid (AR grade), perchloric acid (AR grade), and hydrofluoric acid (AR grade) were purchased from Sinopharm Chemical Reagent Co., Ltd. (Shanghai, China). Hydrogen peroxide (30% purity) and hydroxylamine hydrochloride (AR grade) were purchased from Shanghai Aladdin Co., Ltd. (Shanghai, China). Ultrapure water from the UPT-I-5 T system of Shanghai Yetuo Technology Co., Ltd. (Shanghai, China) was used for adsorption experiments.

### Preparation of the remediation materials

The preparation of humic acid is composed of sodium humate and wood vinegar in a mass ratio of 1:3. At this time, the sodium humate is acidified by wood vinegar to become black-brown precipitate humic acid. Biochar-humic acid materials were prepared with a mass ratio of 1:1 biochar and humic acid^36^, the two solutions were uniformly mixed, dried at room temperature, ground and passed through a 0.15 mm sieve to obtain biochar-Humic acid material, namely BC-HA. Biochar powder was added into a certain proportion of sodium humate solution, and the material was filtered and dried to obtain BC-NaHA. Under the same conditions, the biochar-humate sodium material (BC-NaHA) was composed of sodium humate (50.0 wt%) and biochar (50.0 wt%), and the biochar material (BC) was only composed of biochar powder (100 wt%).

The amount of biochar-humic acid added was 0.5 g·kg^−1^, 1.5 g·kg^−1^, 2.5 g·kg^−1^, 3.5 g·kg^−1^, 4.5 g·kg^−1^ and 5.5 g·kg^−1^ , and these six experimental groups are denoted as BC-HA-1, BC-HA-2, BC-HA-3, BC-HA-4, BC-HA-5 and BC-HA-6. The same amount of biochar-sodium humate experimental groups were denoted as BC-NaHA-1, BC-NaHA-2, BC-NaHA-3, BC-NaHA-4, BC-NaHA-5 and BC-NaHA-6. The same amount of biochar experimental groups are denoted as BC-1, BC-2, BC-3, BC-4, BC-5 and BC-6. Contaminated soil without adding any material after aging was used as the control group, denoted as CK. Each treatment was repeated three times to ensure the accuracy of the experimental data. The above materials were evenly mixed with the contaminated soil and reacted for 60 days.

### Adsorption of different forms of nickel in soil by biochar-based materials

1. The content of available nickel in soil extracted by CaCl_2_.
  After culturing the remediation materials and polluted soil in the same environment for 60 days, the above experimental group and control group were sampled by the five-point sampling method. The content of available nickel in 1.0 g soil samples before and after treatment with three remediation materials was extracted with 0.01 M CaCl_2_ (1:10 w/v)^37^.

(2) Four chemical forms of nickel in soil were extracted by BCR sequential extraction.

Soil samples treated with BC-HA-1, BC-HA-2, BC-HA-3, BC-HA-4, BC-HA-5, BC-HA-6 were air-dried and ground, and passed through a 0.125 mm sieve. The content of four chemical forms of nickel in contaminated soil was extracted by BCR sequential extraction method, as shown in Table 1.

**Table 1:** BCR sequential extraction process for Ni of soil samples.

Step	Fraction	Extraction agent	Reaction conditions
1	Exchangeable fraction	0.11 M CH_3_COOH	16 h, 25 °C
2	Reducible fraction	0.1 M NH_4_OH·HCl	16 h, 25 °C
3	Oxidizable fraction	8.8 M H_2_O_2_ 1 M CH_3_COONH_4_	85 °C for 1 h and room temperature for 16 h
4	Residual fraction	HNO_3_:HClO_4_:HF = 3:1:1(v/v)	Under microwave digestion

All metal extracts and filtrate concentrations were determined by atomic absorption spectrometer (AAS). Three replicates were set up for each sample, and the average value is displayed.

### Sorption properties of remediation materials

#### Adsorption kinetics

A Ni solution was prepared with an initial concentration of 200 mg·L^−1^ (with 0.01 mol·L^−1^NaNO_3_ as the background) in 1000 mL-beakers. Under the temperature of 25 °C and the initial pH of 5, stirring with a thermostatic magnetic stirrer, 0.5 g of BC-HA is added into the Ni(II) solution. At the adsorption time of 5, 10, 30, 60, 120, 180, 240, 360, 480, 600, 720, 1440 min, the solution samples are quickly drawn with a pipette to pass through a 0.22 μm filter membrane. The concentration of nickel in the filtrate are determined to calculate their adsorption capacity by an AAS.

### Adsorption isotherm

0.2 g BC-HA is added to a 50 mL polyethylene centrifuge tube, then 20 mL Ni(II) solution (with 0.01 mol·L^−1^NaNO_3_ as the background) is added to the tube. After that, the concentrations of the nickel solutions are set as 10, 20, 40, 60, 80, 100, 120, 150, 180, 200 mg·L^−1^. The centrifuge tube is oscillated at 200 r·min^−1^ for 24 h in a shaking incubator with a constant temperature of 15 °C, 25 °C and 35 °C, respectively. After oscillation, the sample is centrifuged using a centrifuge at 8000 r·min^−1^ for 10 min. The supernatant solution is passed through a 0.22 μm filter membrane, and the concentration of nickel in the filtrate is measured by the AAS, and the adsorption capacity of BC-HA is calculated.

### Nickel ion adsorption under different pH conditions

0.2 g BC-HA is added to a 50 mL polyethylene centrifuge tube, then 20 mL Ni(II) solution (with 0.01 mol·L^−1^NaNO_3_ as the background) is added to the tube. After that, the initial pH of the nickel solutions are set as 2, 3, 4, 5, and 6. The centrifuge tube is oscillated at 200 r·min^−1^ for 24 h in a shaking incubator with a constant temperature of 25 °C. After oscillation, the sample is centrifuged using a centrifuge at 8000 r·min^−1^ for 10 min. The supernatant solution is passed through a 0.22 μm filter membrane, and the concentration of nickel in the filtrate is measured by the AAS, and the adsorption capacity of BC-HA is calculated.

### FT-IR, SEM/EDS, BET and XPS measurements

All BC-HA, BC-NaHA, BC and their immobilization samples are rinsed three times with ultrapure water before and after immobilize metal ions to remove any physisorbed metal ions and dried under vacuum overnight before all measurements^38^.

Using the KBr disc technique, the KBr and the sample were uniformly ground and pressed into thin slices in a 200:1 ratio, and the samples were analyzed by a VECTOR-22 Fourier transform infrared spectrometer (Bruker, Germany). A Q45 SEM(FEI, USA) was used to characterize the micromorphology of all BC-HA, BC-NaHA, BC and their immobilization samples. Appropriate amount of samples to be tested are uniformly pasted on the conductive adhesive surface and fixed on the sample table. After spraying gold, the instrument is used for testing. BET analysis is run using a Surface Area and Porosity Analyzer (MICROMERITICS ASAP 2460, USA). BC-HA, BC-NaHA, and BC samples were vacuum-dried at 120 °C for 5 h before testing. XPS analysis is run using an XPS spectrometer (AXIS Supra type, Kratos, UK). The spectra of all BC-HA, BC-NaHA, BC and their immobilization samples are obtained over the range of 0 to 1200 eV, with a slit width of 1.9 mm into the analyzer and an energy of 300 eV. The binding energy is calibrated using the C 1 s peak as 284.60 eV.

### Data analysis

#### Adsorption capacity

The adsorption amount of metals is calculated according to the following formula:

1 $$Q_{e} = (C_{0} - C_{e} ) \times \frac{V}{W}$$

where *Q*_*e*_ is the adsorption amount of metals, mg·g^−1^; *C*_*0*_ and *C*_*e*_ are the initial and equilibrium concentration of Ni(II), mg·L^−1^; *V* is the volume of the solution, mL; *m* is the mass of the soil sample, g.

### Adsorption kinetic

Pseudo-first-order kinetic equation

2 $$Q_{t} = Q_{{\text{e}}} - Q_{{\text{e}}} e^{{( - k_{1} t)}}$$

Pseudo-second-order kinetic equation

3 $$Q_{t} = \frac{{k_{2} Q_{e}^{2} t}}{{1 + k_{2} Q_{e} t}}$$

Elovich dynamics equation

4 $$Q_{t} = K_{{\text{t}}} \ln t + A$$

where *Q*_*e*_ is the adsorption amount at equilibrium, mg·g^−1^; *Q*_*t*_ is the adsorption amount at time of t, mg·g^−1^; *t* is the reaction time, min; *K*_*1*_ is the Pseudo-first-order rate constant, min^−1^; *K*_*2*_ is the Pseudo–second-order rate constant, min^−1^; *A* is the diffusion rate constant, mg·g^−1^; *K*_*t*_ is the reaction rate constant, mg·g·min^−0.5^.

### Adsorption isotherm

Langmuir adsorption isotherm

5 $$Q_{e} = \frac{{K_{L} Q_{m} C_{e} }}{{1 + K_{L} C_{e} }}$$

Freundlich adsorption isotherm

6 $$Q_{e} = K_{F} C_{e}^{\frac{1}{n}}$$

Temkin adsorption isotherm

7 $$Q_{e} = \frac{RT}{{b_{T} }}\ln (K_{T} C_{e} )$$

where *C*_*e*_ is the concentration at adsorption equilibrium, mg·L^−1^; *Q*_*e*_ is the adsorption amount at equilibrium, mg·g^−1^; *Q*_*m*_ is the maximum adsorption amount, mg·g^−1^; *K*_*L*_ is related to the size of the adsorption energy Constant, L·mg^−1^; *K*_*F*_ is a constant related to the adsorption strength, mg·g^−1^; *n* is the heterogeneity of the adsorbent. The factor, when n > 1, indicates that there is a strong force between the adsorbate and the adsorbent; *R* is the gas constant, which is 8.314 J·mol^−1^·K^−1^; *T* is the Kelvin temperature, K; *b* is the Temkin constant, J·mol^−1^; *K*_*T*_ is the equilibrium constant at the maximum adsorption capacity, L· mg^−1^.

All data are analyzed by the single-factor analysis of variance (one-way ANOVA) using SPSS24.0 (SPSS Inc. Chicago, USA), the data is the mean ± standard deviation, and lowercase letters indicate significant differences between different groups under the same percentage (p < 0.05).

## Results and discussion

### The effect of biochar-based materials on the availability of nickel in soil

The availability of metals generally refers to the degree of absorption, accumulation or toxicity of metals in the ecological environment^39^. The distribution of heavy metals in various chemical forms is affected by soil aging, and the type of metal determines the degree of redistribution of metal forms. The redistribution of heavy metal forms in soil is characterized by rapid preservation at the beginning and gradual transition later^40^. In this study, CaCl_2_ was used to extract the available content of nickel in soil.

**Figure 1:**
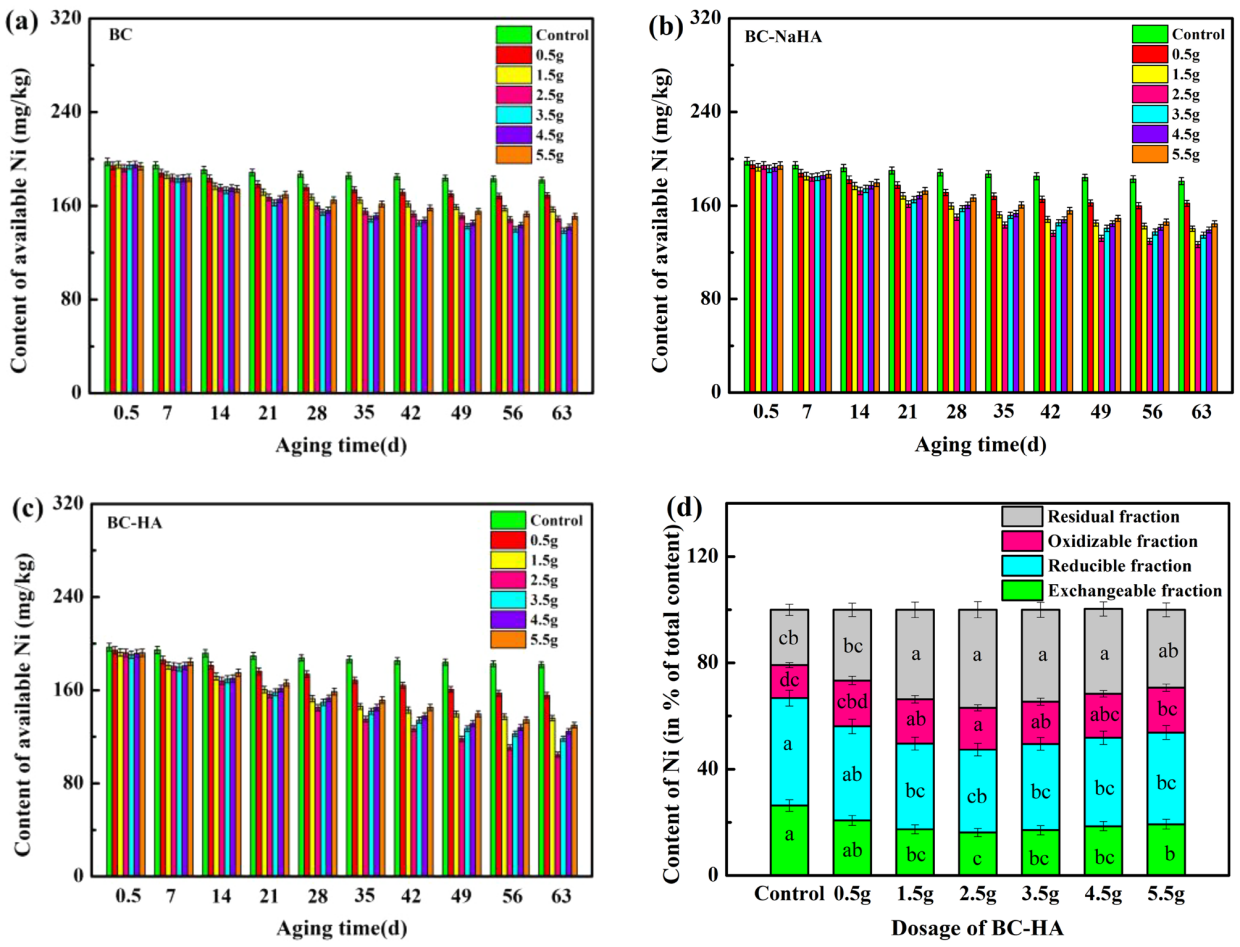
The effect of (**a**) BC, (**b**) BC-NaHA, (**c**) BC-HA with different addition amounts with aging time on the available nickel in the soil; (**d**) The effect of the addition of BC-HA on the four chemical components of nickel in the soil.

The effects of different application amounts of BC, BC-NaHA and BC-HA on available nickel in soil with aging time are shown in Fig. 1a-c, while the control group is nickel-contaminated soil without any remediation materials. It can be seen from the figure that with the increase of aging time, compared with the control group, after adding BC, BC-NaHA and BC-HA, the content of available nickel in soil decreased to varying degrees. The decreasing degree of soil available nickel content by substances was BC-HA > BC-NaHA > BC.

In Fig. 1a-c, compared with the control group, after aging (63d), 3.5 g·kg^−1^ BC reduced the content of available nickel in soil by 31.27%, and 2.5 g·kg^−1^ BC-NaHA reduced soil available nickel content by 37.16%. 2.5 g·kg^−1^ BC-HA significantly reduced the soil available nickel content by 48.17%, which was 38.85% lower than the control group. The results confirmed that 2.5 g kg^−1^ BC-HA material had the maximum effect on reducing the effective Ni content in soil, which was better than the effect of humus as a cleaning agent to remove Ni in polluted soil reported in the literature (35.4% ~ 46.1%) ^35^. Therefore, it is feasible to desorb and fix nickel in soil with humic acid-modified biochar, and the fixation effect of humic acid and biochar composite on nickel is much greater than that of single material. Compared with BC and BC-NaHA, BC-HA has a stronger capacity for Ni immobilization/stabilization during incubation.

Since the content of available metals in soil cannot describe the distribution of chemical forms of metals in soil in detail, the BCR sequential extraction method was used to study the binding form and migration ability of metals in soil, so as to determine the size of the biological availability of metals. Studies have shown that the accumulation process of metals in soil to plants has a good correlation with the bioavailable concentration of metals in soil^39^.

Figure 1d showed the distribution of nickel in soil supplemented with BC-HA after aging culture (63d). It can be seen from the figure that the acid-soluble content of nickel was significantly reduced, which was attributed to the adsorption and precipitation of metals on the adsorbent surface, which was consistent with the literature results^41^. Compared with the control group, the exchangeable and reducible components of Ni(II) in the soil treated with 2.5 g BC-HA decreased by 10.11% and 9.29%, respectively, while the oxidizable and residual components of Ni(II) increased by 3.30% and 16.10%, respectively. The reason was attributed to the combination of Ni(II) with carbonate and Fe/Mn oxides after the addition of BC-HA material, resulting in a significant reduction of Ni content in weak acid extraction and reduction states, forming insoluble stable complexes^42^. The increased content of nickel oxidizable state components can be attributed to the formation of nickel complexes with organic functional groups present in the sorbent^43,44^. In addition, the largest immobilization of Ni in soil was the transformation from the acid-soluble fraction of Ni to residual Ni^44,45^. Therefore, the conversion of acid-soluble nickel components into residual components is an effective way for nickel to be solidified in soil. The results showed that when BC-HA was added into soil, the weak acid extract and reduced nickel components in soil were transformed into oxidizable and residual components, which were not easy to be absorbed by plants and had low environmental mobility. Therefore, BC-HA was a good curing agent for stabilizing and repairing nickel-contaminated soil.

### Adsorption kinetics

The experimental data were fitted using the Pseudo-first-order kinetic model (Eq. 2), the Pseudo-second-order kinetic model (Eq. 3) and the Elovich (Eq. 4) model. Generally, the Pseudo-first-order kinetic model considers the adsorption mechanism to be physical adsorption, the Pseudo-second-order model considers the adsorption mechanism to be monolayer adsorption through chemisorption, and the Elovich kinetic model considers chemisorption on a heterogeneous surface.

**Figure 2:**
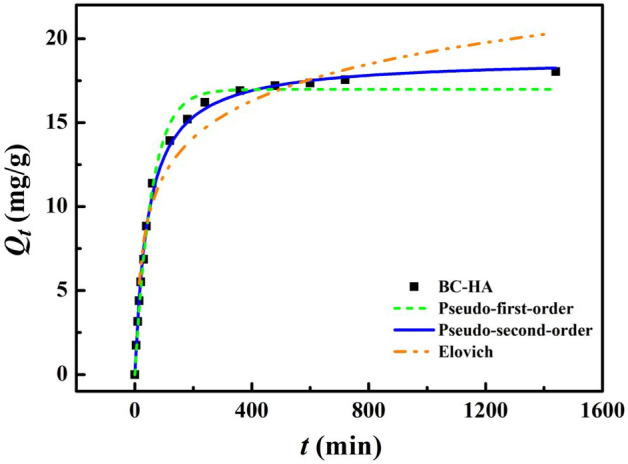
Three kinetic models for adsorption of Ni(II) ions by BC-HA.

The relationship between the adsorption amount of Ni(II) adsorbed by BC-HA and the adsorption time is shown in Fig. 2. The adsorption capacity of BC-HA for Ni(II) increased rapidly in the first 240 min, was in the slow adsorption stage in 240 ~ 720 min, and reached the equilibrium state in 1440 min, with the equilibrium adsorption capacity of 18.04 mg/g. The reason is that sufficient adsorption sites are provided on the surface of BC-HA in the initial stage, and Ni(II) occupy the sites quickly. However, with the progress of the adsorption process, the active sites on the surface of BC-HA were reduced, and the adsorption rate gradually decreased. With the increase of the adsorption time, the amount of Ni(II) adsorbed by BC-HA increased continuously. The reason was that the driving force of the concentration difference existing when the solution was at a high concentration made the Ni(II) sneak into the interior of BC-HA, expanding the Utilization of adsorption sites. Since the kinetic model assumes that the adsorption amount increases in the initial adsorption stage, the adsorption rate slows down as the adsorption process progresses, and finally approaches the adsorption equilibrium. Therefore, the above results were consistent with the kinetic model.

The pseudo-first-order kinetic equation was well fitted (R^2^ = 0.992) (Table 2), indicating that the adsorption process of BC-HA on Ni (II) may be affected by surface physical adsorption. However, The adsorption kinetic data of Ni(II) onto BC-HA could be well-described by the Pseudo-second-order model, showing high regression coefcients of R^2^ = 0.997 (Table 2). Meanwhile, the theoretical *Q*_*e*_ values ftted by the Pseudo-second-order model were 18.82 mg/g for Ni(II) ion, which was very close to the experimental result of 18.04 mg/g (Table 2). The better ftting results of the Pseudo-second-order model than the Pseudo-first-order and Elovich model indicated that chemical adsorption was mainly responsible for the immobilized of Ni(II) ion by BC-HA^46^.

**Table 2:** Fitting parameters of adsorption kinetics of BC-HA adsorption of Ni(II) ions.

Type		Linear model
Pseudo-first-order	Q_e_(mg·g^−1^)	16.98
	K_1_(min^−1^)	0.018
	R^2^	0.992
Pseudo-second-order	Q_e_(mg·g^−1^)	18.82
	K_2_(g·mg^−1^·min^−1^)	0.001
	R^2^	0.997
Elovich	A(mg·g^−1^)	− 2.567
	K_t_(mg·g·min^−0.5^)	3.150
	R^2^	0.950

### Adsorption isotherms

**Figure 3:**
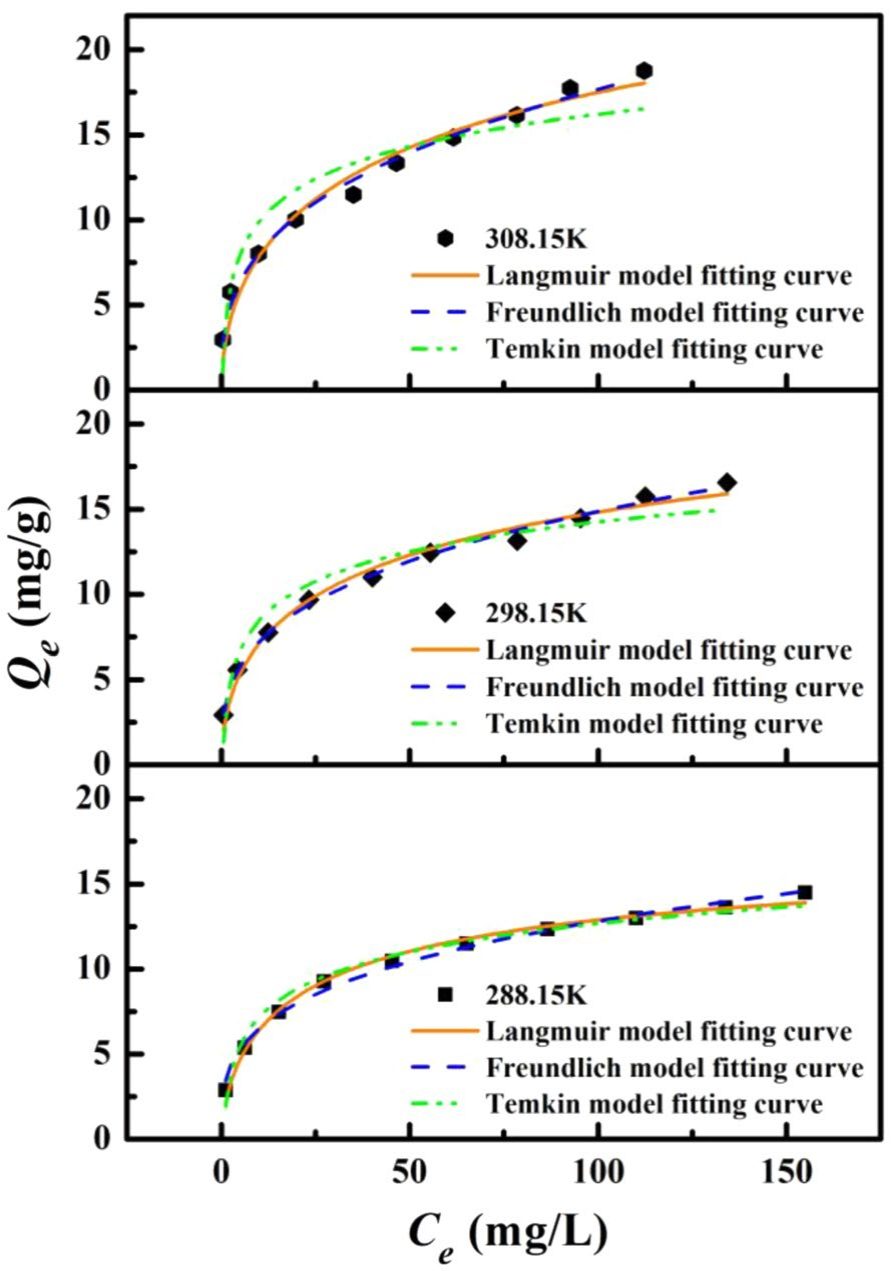
Three isotherm models of adsorption of Ni(II) ion by BC-HA at temperatures of 288.15 K, 298.15 K and 308.15 K.

The Langmuir (Eq. 5), Freundlich (Eq. 6) and Temkin (Eq. 7) isotherm models widely adopted for evaluation of adsorption behaviors were used to fit the isotherm data (Fig. 3). The ftting parameters for each isotherm model are compiled in Table 3. In general, the Langmuir model assumes that all sites have the same affinity for pollutants, and when the solid surface is saturated, a monolayer adsorption is formed; the Freundlich model assumes that the adsorption of the molecular layer occurs on a non-uniform surface, and the adsorption mechanism is Multilayer adsorption; the Temkin isotherm model, which assumes that the heat of adsorption (a function of temperature) of all molecules in the adsorption layer decreases linearly with increasing coverage area, mainly describes the chemisorption process as an electrostatic interaction^47^.

**Table 3:** Fitting parameters of adsorption thermodynamics for BC-HA adsorption of Ni(II).

Type		288.15 K	298.15 K	308.15 K
Langmuir	Q_m_ (mg·g^−1^)	14.60	16.73	19.78
	K_L_ (L·mg^−1^)	0.121	0.101	0.082
	R^2^	0.989	0.983	0.968
Freundlich	K_F_ (L·g^−1^)	3.306	3.476	3.672
	n	3.400	3.170	2.933
	R^2^	0.991	0.995	0.990
Temkin	K_T_ (L·mg^−1^)	1.968	2.853	3.665
	B (J·mol^−1^)	998.9	983.9	932.9
	R^2^	0.977	0.932	0.897

As can be seen from Fig. 3, the initial concentration of the solution is the same, and the increase of temperature is conducive to the increase of the adsorption capacity of Ni(II) by BC-HA, and eventually tends to the adsorption equilibrium. The Langmuir model has a good fit, indicating that Ni(II) may be physically adsorbed on the BC-HA material by the monolayer. The Freundlich model has the greatest fitting degree, indicating that the adsorption of Ni(II) was mainly multi-layer adsorption on the surface of the adsorbent, which was consistent with the results reported in the literature^47,48^^.^ The maximum adsorption amount of Ni(II) obtained by the Langmuir model is 19.78 mg/g (Table 3), which was better than the maximum adsorption amount of Ni (12.41 mg/g) by rice husk humic acid in the literature^49^. The results showed that the added BC-HA material with WV has an enhanced ability to adsorb Ni(II).

At temperatures of 288.15 K, 298.15 K and 308.15 K, the K_L_ values in the Langmuir model ranged from 0.082 to 0.121 L/mg (Table 3).The K_L_ value is related to the adsorption capacity. The larger the K_L_ value, the stronger the adsorption capacity, indicating that the increase of temperature was beneficial to reduce the interaction between the adsorbent and the solvent surface and promote the adsorption of Ni(II) on BC-HA. The n value in the Freundlich model varies from 2.933 to 3.400 (Table 3), and was between 1 and 10, indicating that there was a strong force between the adsorbate and the adsorbent^50^. The results showed that BC-HA had a strong ability to adsorb Ni(II) ion.

### Effects of solution pH on adsorption

**Figure 4:**
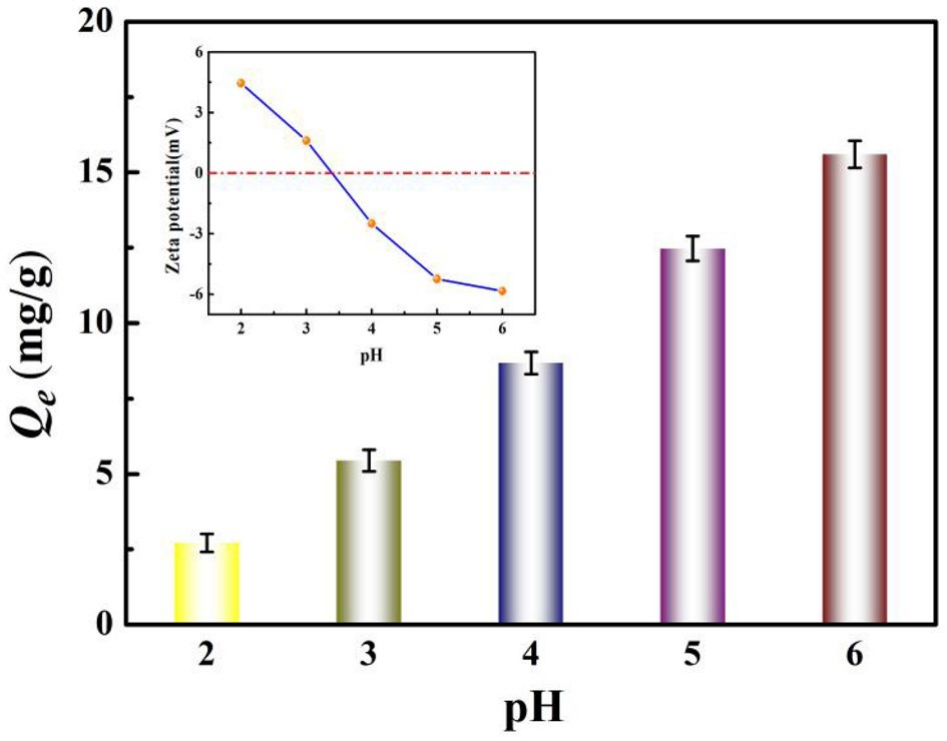
The effect of solution pH on the adsorption of Ni(II) by BC-HA.

Figure 4 shows the variation of Ni(II) ion adsorption onto BC-HA with diferent initial solution pH. Solution pH significantly affects the adsorption capacity of heavy metals by affecting the complexation behavior of surface-active functional groups (hydroxyl, carboxyl, and amino groups)^51^. When the pH value of the solution is greater than 6, nickel ions form hydroxide precipitation on the surface of the adsorbent^52^, so the pH value of the solution was set in the range of 2 to 6 in the experiment.

As can be seen from Fig. 4, the zero point charge pH_ZPC_ value of BC-HA is about 3.4. When pH < pH_ZPC_, a large number of H^+^ ions in the solution occupy the adsorption active site of BC-HA, leading to the increase of electrostatic repulsion between positively charged BC-HA and Ni (II) ions. On the contrary, when pH > pH_ZPC_, the concentration of H^+^ ions decreased, and a large number of negatively charged active groups on the surface of BC-HA were exposed, which promoted the coordination complexation reaction between -OH and -COOH and Ni (II) ions, resulting in the enhanced ability of BC-HA to adsorb Ni (II) ions. The results showed that the maximum adsorption capacity of Ni(II) by BC-HA was 15.60 mg/g at pH 6. Similar results showed that the maximum adsorption of Ni(II) was achieved in the pH range of 5–6^53^. Therefore, all adsorption experiments were performed at pH 6.

### Immobilization mechanism

#### FTIR analysis

**Figure 5:**
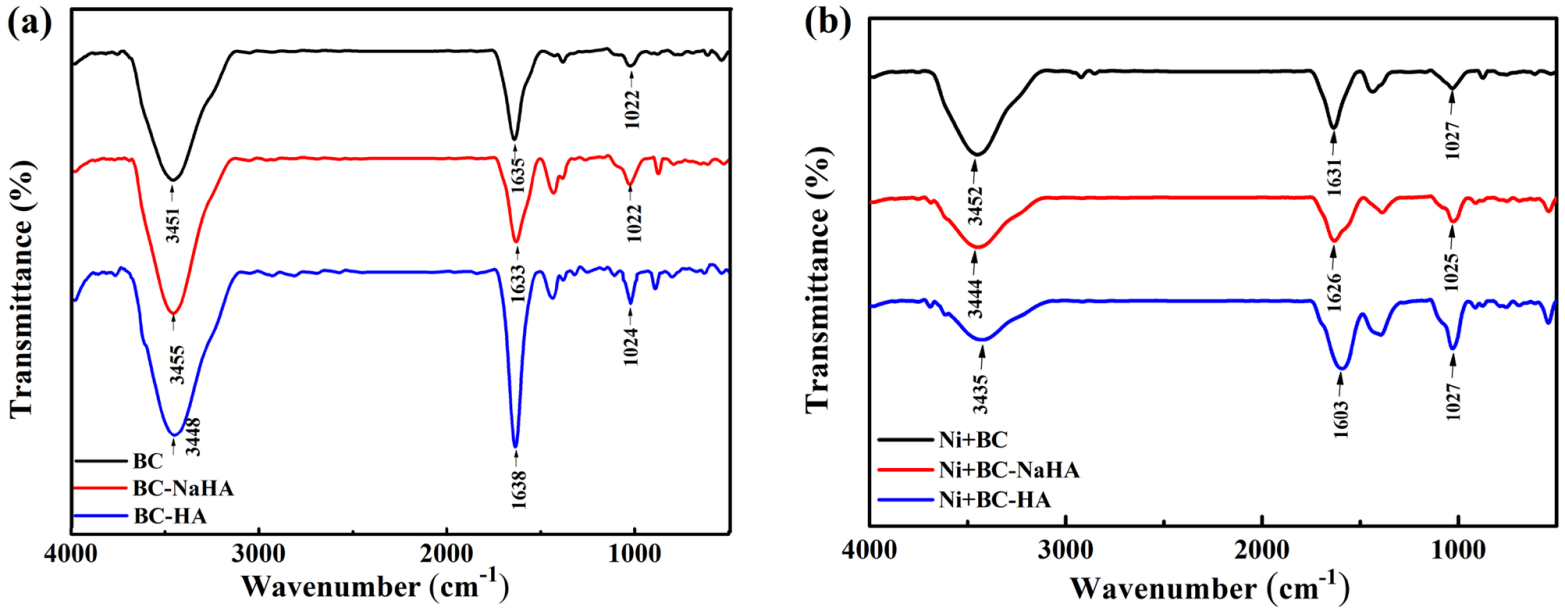
FT-IR spectra of (**a**) BC, BC-NaHA, and BC-HA; (**b**) Ni(II) ions immobilized by BC, BC-NaHA, and BC-HA.

The FTIR spectra of BC, BC-NaHA and BC-HA prior to and after Ni(II) adsorption are presented in Fig. 5a,b. The main absorption bands displayed were assigned as follows^54–56^, the broad peak in the 3448 ~ 3455 cm^−1^ band is the stretching vibration peak of the hydroxyl group (-OH) of phenols; the 1633 ~ 1636 cm^−1^ band is the aromatic C = C and C = O stretching vibration peaks; the 1022–1024 cm^−1^ band region is the stretching vibration peaks of C-O in alcohols, ethers and esters. The FTIR results confirmed that the intensities of the C = C, C = O and C-O peaks of the BC-HA material were much greater than the intensities of the corresponding peaks on the BC and BC-NaHA materials, so the ability of the material to adsorb nickel was BC-HA > BC-NaHA > BC. The results showed that the introduction of WV increased the number of oxygen-containing functional groups on the adsorbent surface and promoted the formation of more metal complexes between BC-HA and Ni(II).

After Ni(II) was adsorbed by BC, BC-NaHA and BC-HA, the stretching vibration peaks of C = O and C–OH were both weakened, indicating that the carboxyl group and hydroxyl group are closely related to the Ni(II) reaction^57^. Compared with BC-NaHA and BC, BC-HA has more hydroxyl and carboxyl groups to react with Ni(II). According to related literature^58^, the complexation of oxygen-containing functional groups on the surface of biochar with Ni was a mechanism for Ni immobilization in soil.

### SEM/EDS analysis

**Figure 6:**
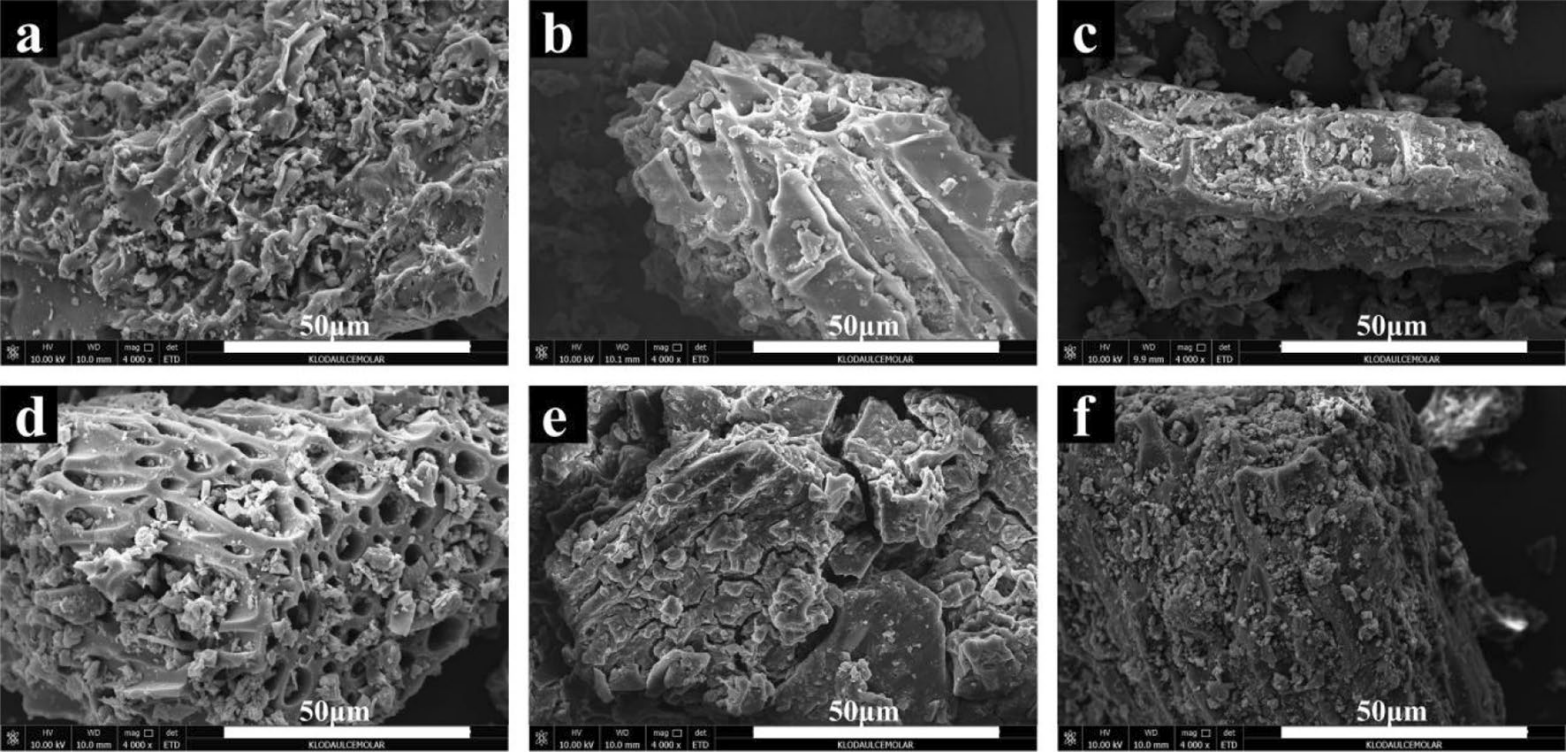
SEM image of (**a**) BC; (**b**) BC-NaHA; (**c**) BC-HA; (**d**) BC + Ni; (**e**) BC-NaHA + Ni (**f**) BC-HA + Ni.

The SEM images of BC, BC-NaHA and BC-HA before and after adsorption of Ni(II) were shown in Fig. 6 at a magnification of 4000 times. It can be seen from the figure that compared with BC and BC-NaHA, BC-HA presents a more complex, rough and porous surface structure with a large number of adsorption pores. Similarly, after adsorption of Ni(II) by BC, BC-NaHA, and BC-HA, the surface of BC-HA exhibited more irregular and heterogeneous morphologies. It was confirmed that the WV in the adsorbent increased the adsorption surface area and porosity, provided more adsorption active sites for Ni(II), and promoted the adsorption of more Ni(II) by BC-HA.

**Figure 7:**
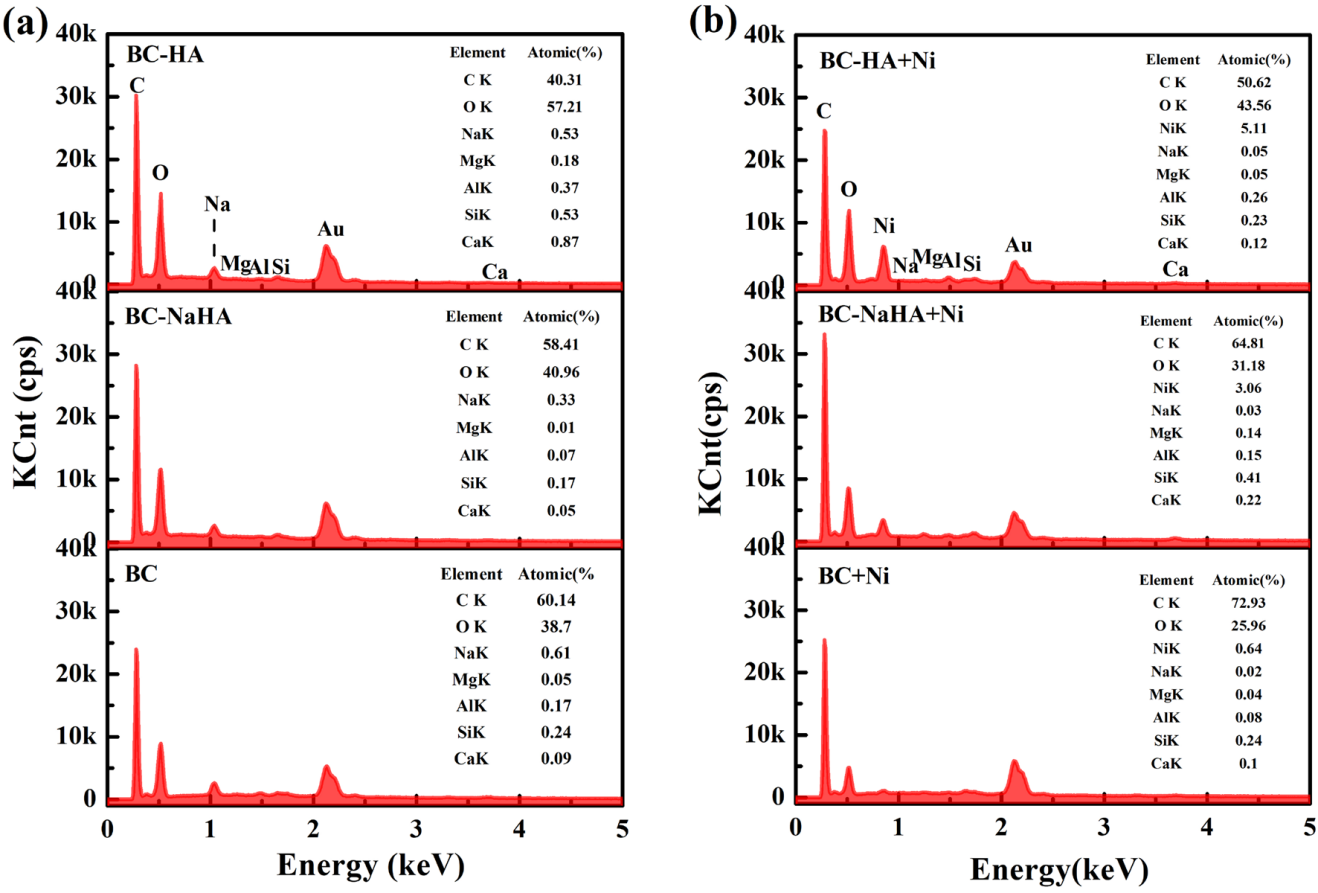
EDS spectrum of (**a**) BC, BC-NaHA, BC-HA; (**b**) BC + Ni, BC-NaHA + Ni, BC-HA + Ni.

The EDS spectra of BC, BC-NaHA and BC-HA before and after adsorption of Ni(II) are shown in Fig. 7a,b. After BC, BC-NaHA and BC-HA adsorbed Ni(II), the percentages of nickel elements in the unit adsorption section were 0.64%, 3.06%, and 5.11%, respectively. Therefore, BC-HA has the greatst adsorption capacity for Ni(II). In addition, the content of other cations such as Na(I), Ca(II), Al(III), Mg(II) changed significantly, which was attributed to ion exchange of Ni(II) with other cations on biochar surface^59^.

Since BC-HA had more oxygen-containing groups than BC-NaHA and BC, the ability of BC-HA to adsorb Ni(II) was stronger than that of BC-NaHA and BC, which was consistent with the FT-IR results. In addition, the above results also confirmed that wood vinegar changed the number of oxygen-containing functional groups in humic acid during the composting process of heavy metal-contaminated soil^60^. The composted humic acid forms insoluble metal complexes with heavy metal ions, thereby reducing the bioavailability of heavy metals in the soil.

### BET analysis

**Figure 8:**
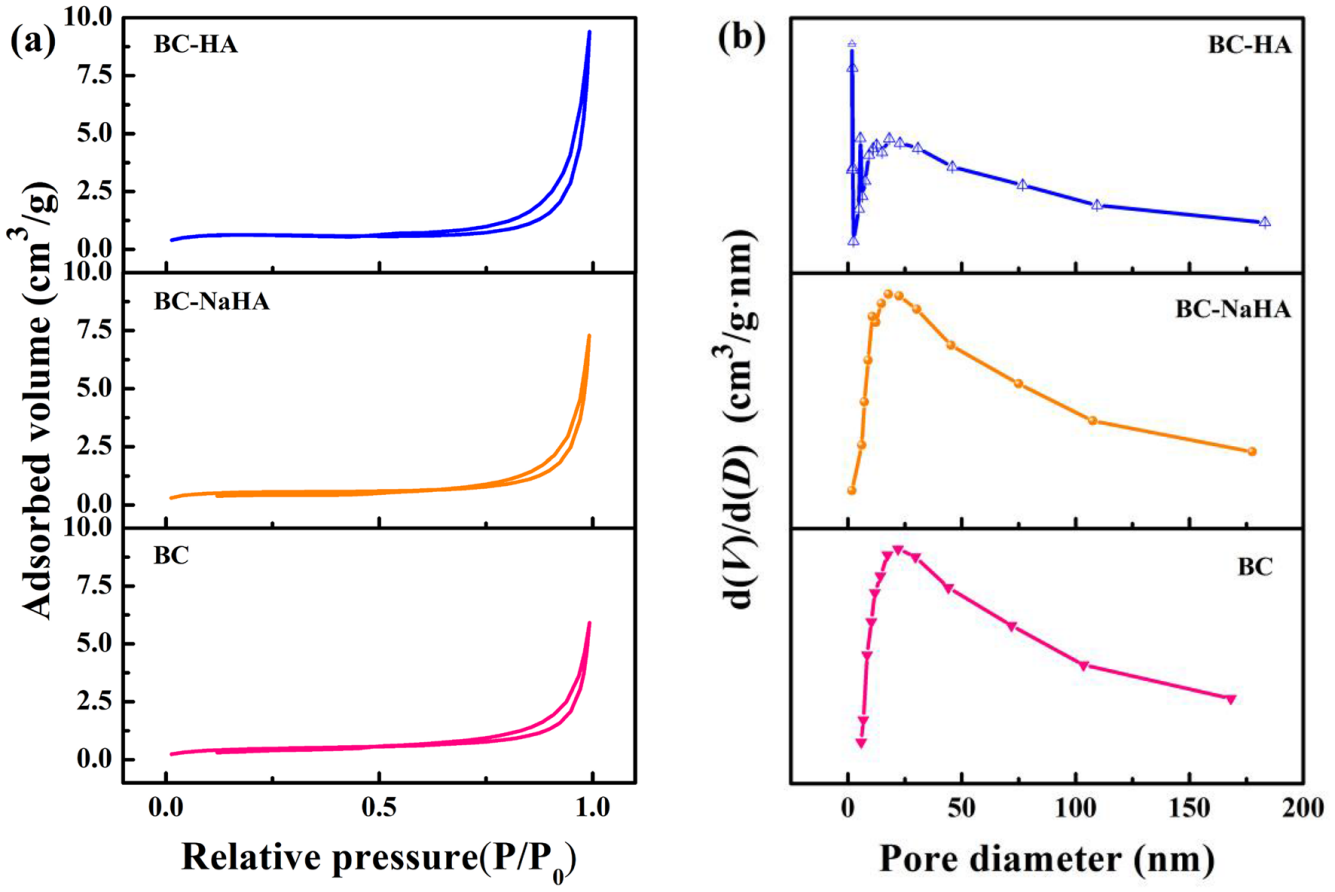
(**a**) N_2_ adsorption–desorption isotherms of BC, BC-NaHA and BC-HA; (**b**) pore size distributions.

The N_2_ adsorption–desorption isotherms and pore size distributions of BC, BC-NaHA and BC-HA at 77.35 K were shown in Fig. 8. According to the International Union of Pure and Applied Chemistry (IUPAC) classification, the isotherms of BC, BC-NaHA and BC-HA were classified as Type IV with a hysteresis loop of H4 type^61^. Type IV isotherm is a characteristic of mesoporous adsorbent materials, and the hysteresis loop is related to narrow slit-like pores in BC-HA^62^. The peak value of pore size distribution of BC-HA was less than 2 nm, indicating that there were a large number of micropores in BC-HA. BC, BC-NaHA and BC-HA all have some pore structure at 2-50 nm, mainly mesoporous structure. In addition, when the relative pressure (P/P_0_) > 0.80, the adsorption capacity of the three materials increased rapidly, which might be responsible for multilayer adsorption^63^.

The specific surface area (SSA) and pore properties of BC, BC-NaHA and BC-HA are listed in Table 4. Compared with BC and BC-NaHA, BC-HA had the largest SSA (1.93 m^2^/g), Barrett Joyner-Halenda (BJH) pore volume (0.015 cm^3^/g), and average pore size (30.18 nm). These results indicated that the pores in BC, BC-NaHA and BC-HA mainly existed in the form of mesopores, and the addition of wood vinegar promoted BC-HA to provide abundant binding active sites for nickel ions. This conclusion also confirms the results of SEM.

**Table 4:** Poreous structure parameters of BC, BC-NaHA and BC-HA.

Samples	S_BET_ (m^2^/g)	V_T_ (cm^3^/g)	D_P_ (nm)
BC	1.61	0.0091	22.63
BC-NaHA	1.85	0.011	24.40
BC-HA	1.93	0.015	30.18

### XPS analysis

**Figure 9:**
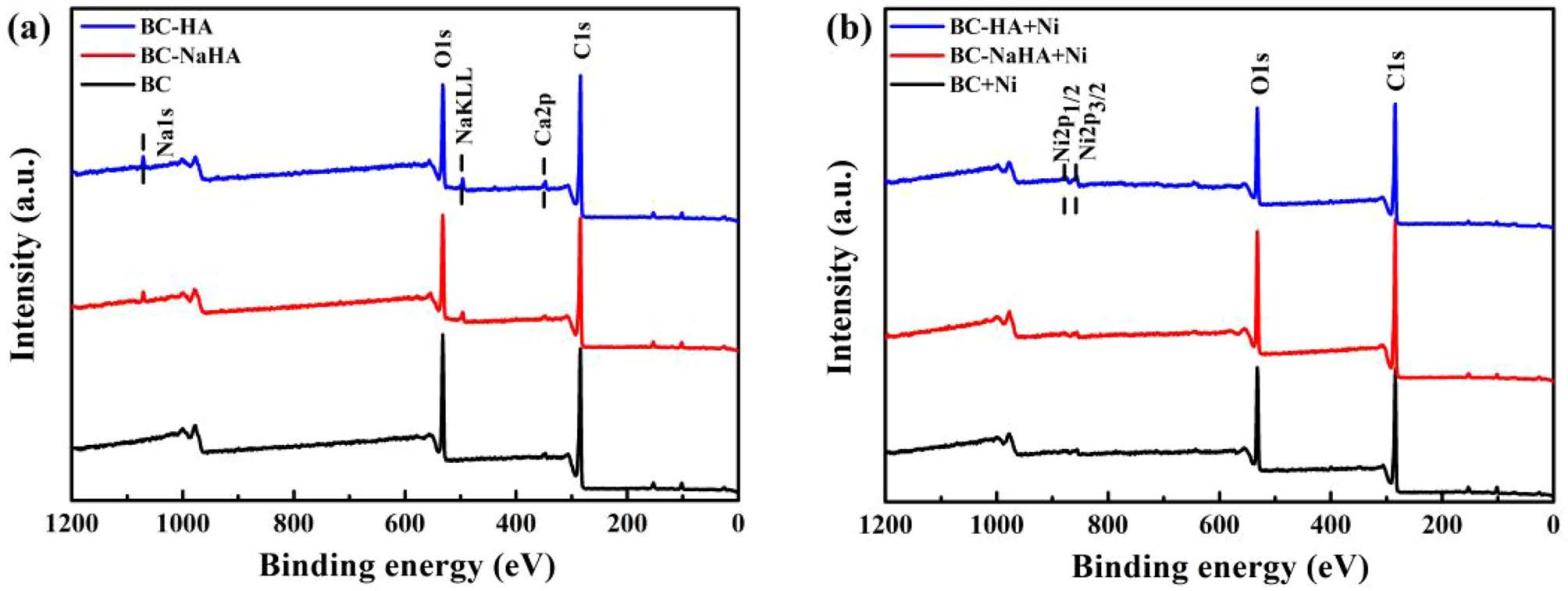
XPS spectra of (**a**) BC, BC-NaHA, BC-HA; (**b**) BC + Ni, BC-NaHA + Ni, BC-HA + Ni.

The XPS spectra of BC, BC-NaHA and BC-HA prior to and after the adsorption of Ni(II) are presented in Fig. 9. After BC-HA and BC-NaHA materials were combined with Ni(II), the peaks of Na1S and Ca2P disappeared, which was attributed to the fact that Ni(II) had replaced Na(I) and Ca(II) to complex with oxygen-containing functional groups of the materials through ion exchange.

**Figure 10:**
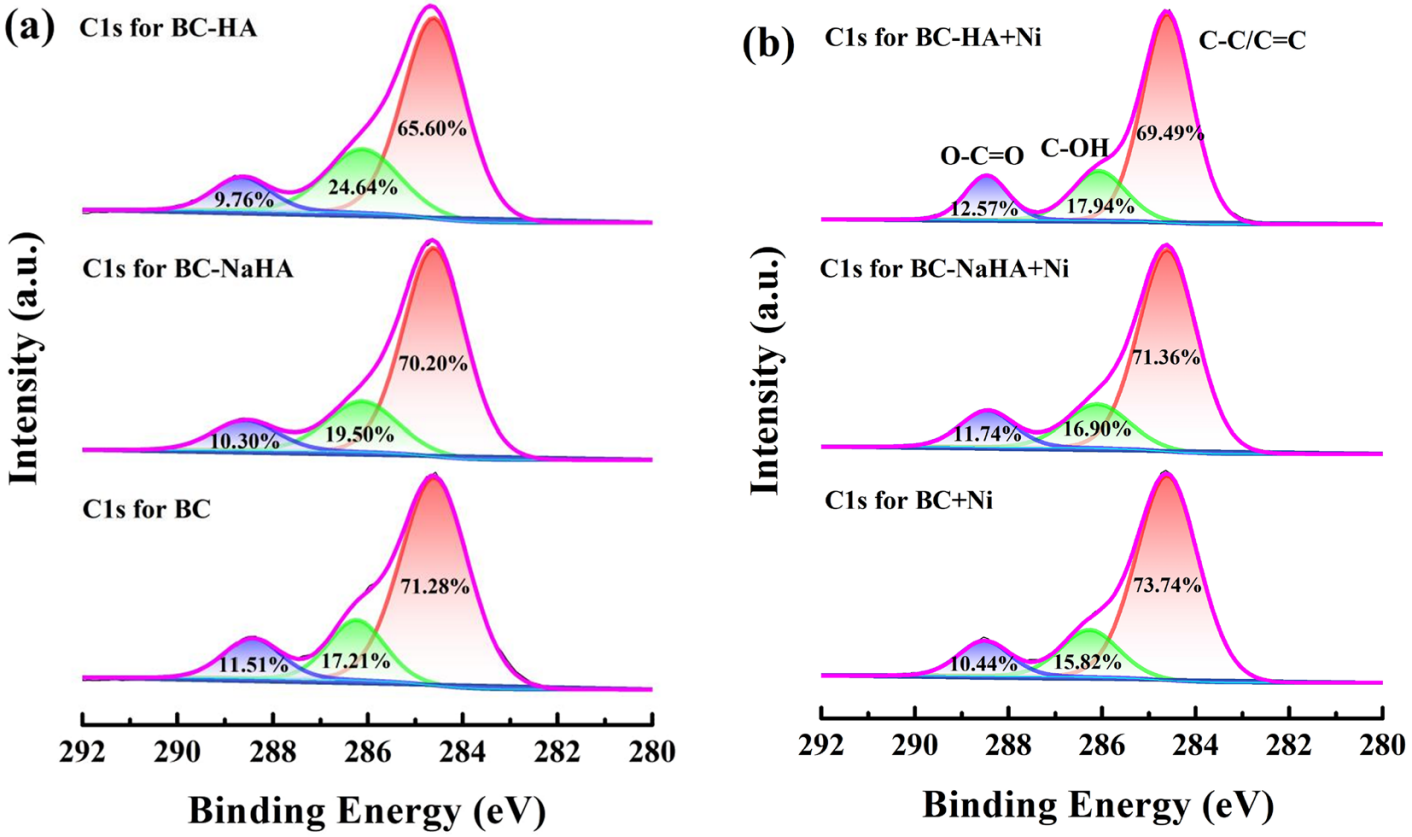
C1s high-resolution spectra (**a**) BC, BC-NaHA and BC-HA; (**b**) BC + Ni, BC-NaHA + Ni, BC-HA + Ni.

The high-resolution spectra of C1s before and after adsorption of Ni(II) by BC, BC-NaHA and BC-HA are shown in Fig. 10. According to related literatures^42,43^, C = C/C–C, C–OH and O-C = O in the C1s spectrum are characteristic peaks at 284.60 eV, 286.10 eV and 288.65 eV, respectively. The contents of C–C/C = C, C–OH, O-C = O in the spectrum before BC adsorption of Ni(II) were 71.28%, 17.21%, and 11.51%, respectively, and after adsorption of Ni(II) were 73.74%, 15.82%, 10.44%, respectively. The contents of C–C/C = C, C–OH, and O-C = O in the spectrum before Ni(II) adsorption by BC-NaHA were 70.20%, 19.50%, and 10.30%, respectively, and the contents of combined Ni(II) were 71.36%, 16.90% and 11.74%, respectively. The contents of C–C/C = C, C–OH, and O-C = O in the spectrum before adsorption of Ni(II) by BC-HA were 65.60%, 24.64%, and 9.76%, respectively, and the contents after adsorption of Ni(II) were 69.49%, 17.94%, and 12.57%, respectively. The above results showed that compared with BC, BC-NaHA and BC-HA, the total oxygen content of BC, BC-NaHA and BC-HA after adsorption of Ni(II) decreased by 2.46%, 1.16% and 3.89%, respectively. In addition, before BC, BC-NaHA and BC-HA adsorb Ni(II), the BC-HA material has the highest total oxygen content among the three materials, followed by BC-NaHA and BC. This was attributed to the modification of NaHA by WV into HA with more oxygen-containing functional groups, which promoted the formation of metal complexes between the hydroxyl and carboxyl groups on BC-HA and Ni(II), making the BC-HA material more conducive to the adsorption of Ni(II).

**Figure 11:**
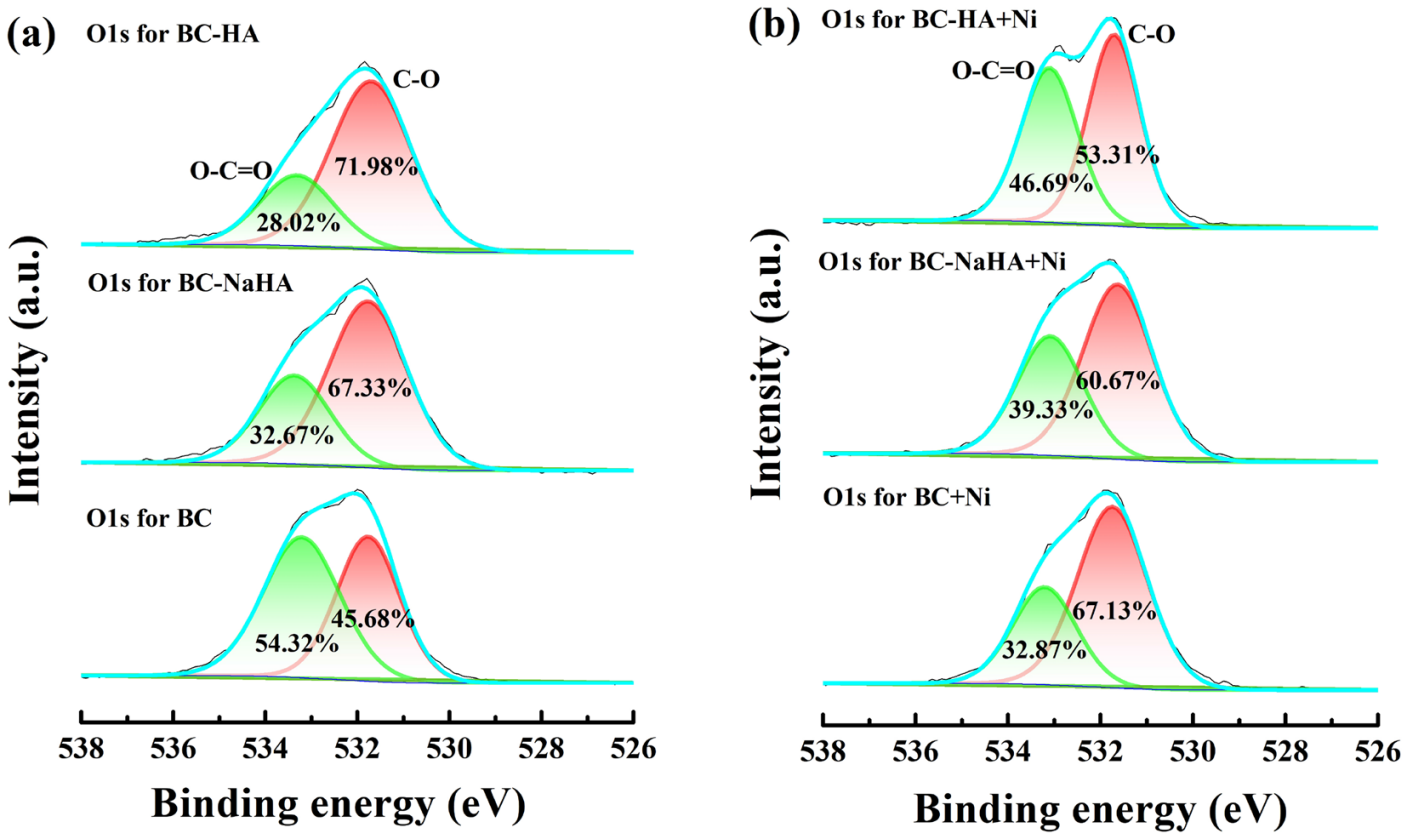
O1s high-resolution spectra (**a**) BC, BC-NaHA and BC-HA; (**b**) BC + Ni, BC-NaHA + Ni, BC-HA + Ni.

The high-resolution O1s spectra of BC, BC-NaHA and BC-HA before and after the adsorption of Ni(II) were shown in Fig. 11. According to related literatures^52,53^, the O1s spectra of BC, BC-NaHA and BC-HA had two binding energy peaks at 531.70 eV and 533.31 eV, which were the characteristic absorptions of C–OH and O-C = O peak, respectively. The contents of C–OH and O-C = O before and after BC adsorption of Ni(II) were 45.68% and 54.32%, 67.13% and 32.87%, respectively. The contents of C–OH and O-C = O before and after the adsorption of Ni(II) by BC-NaHA were 67.33% and 32.67%, 60.67% and 39.33%, respectively. The contents of C–OH and O-C = O before and after adsorption of Ni(II) by BC-HA were 71.98% and 28.02%, 53.31% and 46.69%, respectively. The above results confirmed that the hydroxyl or carboxyl group on the adsorbent reacted with Ni(II) to form metal complexes, which reduced the migration and transformation of heavy metal ions in the environment^45^ The results have proved that more carboxyl groups were bound to Ni(II) in BC materials, while more Ni(II) were bound to hydroxyl groups on the surface of BC-NaHA and BC-HA materials, which was the same as the results reported in the literature^46^. This result was consistent with the results of FT-IR, SEM, EDS, BET analysis.

### Summary of mechanism of adsorption of nickel ions by BC-HA

**Figure 12:**
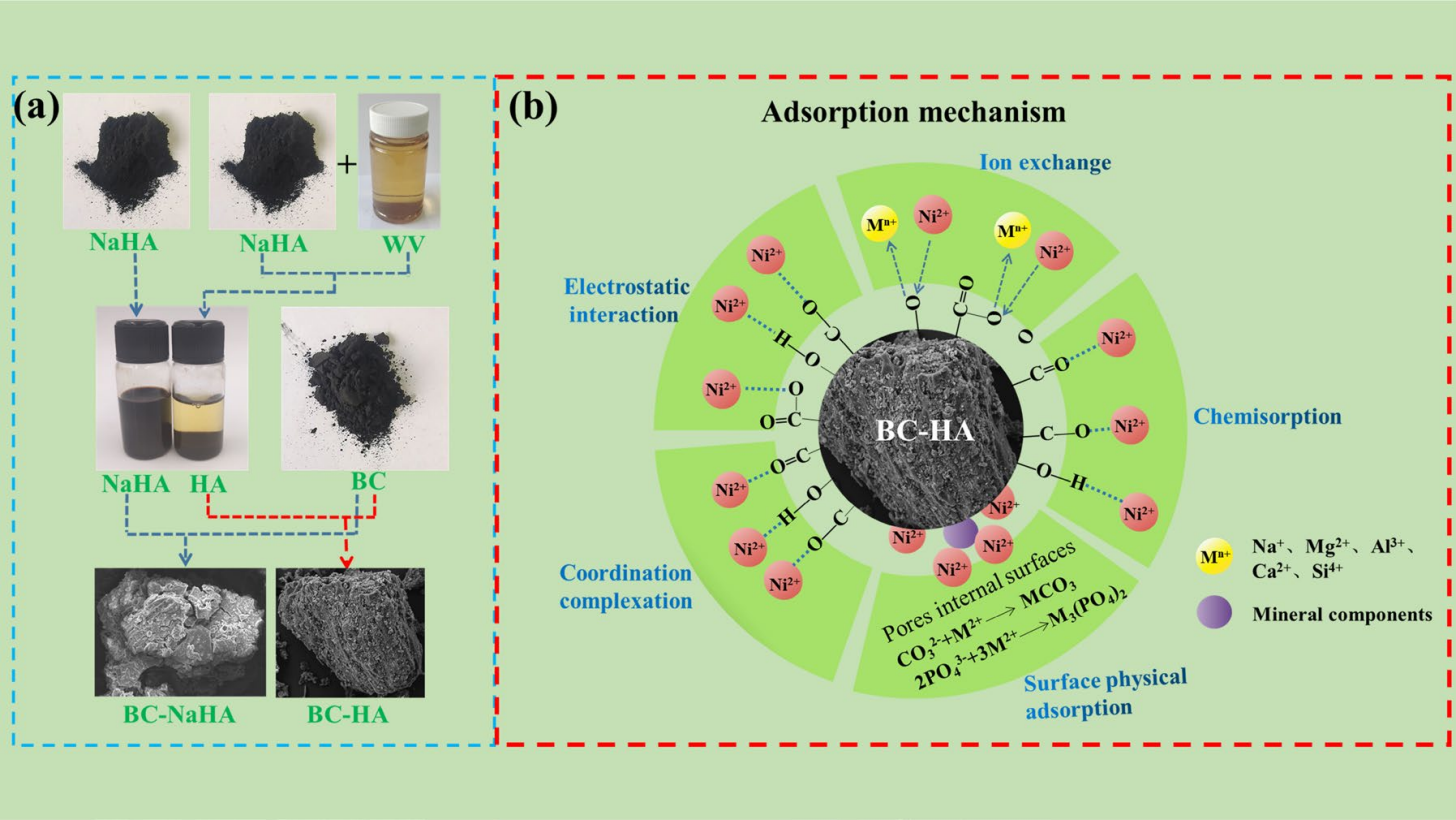
(**a**) Preparation steps of BC-HA material, (**b**) Mechanism Diagram of Ni(II) adsorption by BC-HA.

The preparation procedure of BC-HA material and its adsorption mechanism for nickel ions are shown in Fig. 12. The addition of BC-HA effectively reduced the migration and transformation capacity of nickel in soil and environmental risks, which can be attributed to the coordination reaction between organic functional groups in the adsorbent and Ni(II) to form insoluble and stable complexes. From the results of adsorption kinetics, it can be concluded that the process of adsorption of Ni(II) by BC-HA may be affected by surface physical adsorption, but the adsorption mechanism was mainly chemical adsorption. The adsorption thermodynamic results showed that the adsorption mechanism of Ni(II) by BC-HA was multi-layer adsorption, and the increase of temperature was beneficial to the adsorption reaction. At different pH values, the mechanism of Ni(II) adsorption by BC-HA was mainly electrostatic interaction and coordination. FT-IR results showed that the adsorption mechanism mainly included ion exchange and coordination. SEM/EDS and BET results showed that the adsorption mechanism included physical adsorption, chemical adsorption, ion exchange and coordination. The results of XPS characterization showed that the adsorption principle was mainly ion exchange and coordination. The mechanism of BC-HA adsorption of nickel ions includes surface physical adsorption, chemical adsorption, electrostatic interaction, ion exchange and coordination.

## Conclusions

A) Biochar combined with sodium humate-wood vinegar enhances the ability of soil to absorb and solidify nickel ions, and reduces the bioavailability of nickel ion; Applying 2.5 g of BC-HA material, the exchangeable and reducible content were significantly reduced, and the content of oxidizable components and residues are significantly increased; the reduction rate of available nickel content reached 48.17%.
B) The chemisorption of Ni(II) ions by BC-HA conforms to the Pseudo-second-order kinetic model. The adsorption curve of BC-HA adsorption of Ni(II) shows the Freundlich model for multilayer adsorption. It has better adsorption performance when the pH value is 6. The heterogeneous structure increases the pore structure, increases the adsorption surface area, and has more active sites. The adsorption mechanisms include surface physical adsorption, chemical adsorption, electrostatic interaction, ion exchange, and coordination.
C) This work provides scientific guidance for the development of low-cost, widely applicable soil remediation materials and application techniques, which can be used in soil contaminated by potential for toxic elements.

## Data Availability

All data generated or analysed during this study are included in this published article.
