# Publisher Correction: Effects of biochar-based materials on nickel adsorption and bioavailability in soil

**DOI:** 10.1038/s41598-023-35684-6

**Published:** 2023-05-25

**Authors:** Weichun Gao, Wei He, Jun Zhang, Yifei Chen, Zhaoxin Zhang, Yuxiao Yang, Zhenjia He

**Affiliations:** 1 Shaanxi Provincial Land Engineering Construction Group Co., Ltd., Xi’an, 710075 China; 2 School of Water Resources and Hydropower, Xi’an University of Technology, Xi’an, 710048 China; 3 Institute of Land Engineering and Technology, Shaanxi Provincial Land Engineering Construction Group Co., Ltd., Xi’an, 710075 China; 4 College of Chemistry and Chemical Engineering, Shaanxi Key Research Laboratory of Chemical Additives, Shaanxi University of Science and Technology, Xi’an, 710021 China

Correction to: *Scientific Reports* 10.1038/s41598-023-32502-x, published online 11 April 2023

The original version of this Article contained errors in Figure 7, where panel (**a**) was a duplication of panel (**b**).

The original Figure 7 and accompanying legend appear below.

**Figure 7:**
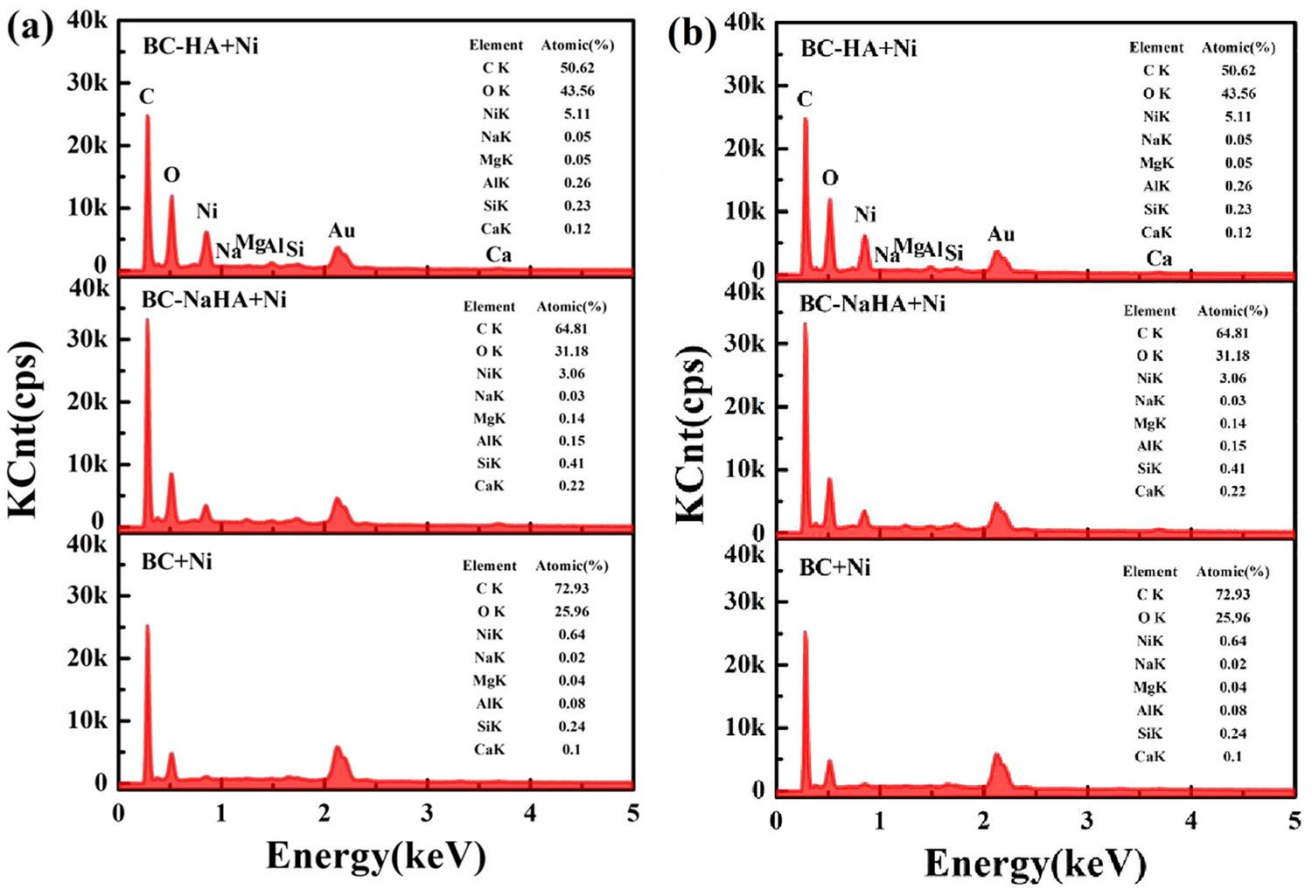
EDS spectrum of (**a**) BC, BC-NaHA, BC-HA; (**b**) BC + Ni, BC-NaHA + Ni, BC-HA + Ni.

The original Article has been corrected.

